# Precision immunointerception of EGFR-driven tumorigenesis for lung cancer prevention

**DOI:** 10.3389/fimmu.2023.1036563

**Published:** 2023-02-17

**Authors:** Jing Pan, Donghai Xiong, Qi Zhang, Katie Palen, Robert H. Shoemaker, Bryon Johnson, Shizuko Sei, Yian Wang, Ming You

**Affiliations:** ^1^ Center for Cancer Prevention, Houston Methodist Cancer Center, Houston Methodist Research Institute, Houston, TX, United States; ^2^ Cancer Center and Department of Pharmacology & Toxicology, Medical College of Wisconsin, Milwaukee, WI, United States; ^3^ Department of Microbiology and Immunology, Medical College of Wisconsin, Milwaukee, WI, United States; ^4^ Chemopreventive Agent Development Research Group, Division of Cancer Prevention, National Cancer Institute, Bethesda, MD, United States

**Keywords:** precision immunoprevention, immunointerception, EGFR mutation, peptide vaccine, MHC class II, T cell

## Abstract

Epidermal growth factor receptor (EGFR) mutations occur in about 50% of lung adenocarcinomas in Asia and about 15% in the US. EGFR mutation-specific inhibitors have been developed and made significant contributions to controlling EGFR mutated non-small cell lung cancer. However, resistance frequently develops within 1 to 2 years due to acquired mutations. No effective approaches that target mutant EGFR have been developed to treat relapse following tyrosine kinase inhibitor (TKI) treatment. Vaccination against mutant EGFR is one area of active exploration. In this study, we identified immunogenic epitopes for the common EGFR mutations in humans and formulated a multi-peptide vaccine (E^mut^ Vax) targeting the EGFR L858R, T790M, and Del19 mutations. The efficacy of the E^mut^ Vax was evaluated in both syngeneic and genetic engineered EGFR mutation-driven murine lung tumor models with prophylactic settings, where the vaccinations were given before the onset of the tumor induction. The multi-peptide E^mut^ Vax effectively prevented the onset of EGFR mutation-driven lung tumorigenesis in both syngeneic and genetically engineered mouse models (GEMMs). Flow cytometry and single-cell RNA sequencing were conducted to investigate the impact of E^mut^ Vax on immune modulation. E^mut^ Vax significantly enhanced Th1 responses in the tumor microenvironment and decreased suppressive Tregs to enhance anti-tumor efficacy. Our results show that multi-peptide E^mut^ Vax is effective in preventing common EGFR mutation-driven lung tumorigenesis, and the vaccine elicits broad immune responses that are not limited to anti-tumor Th1 response.

## Introduction

Lung cancer is the second most common cancer and the leading cancer killer, accounting for 25% of all cancer-related deaths (1). The majority of lung cancers (about 85%) are non-small cell lung cancer (NSCLC), and epidermal growth factor receptor (EGFR) mutations are the most frequent driver mutation in lung cancer, occur in around 50% of Asia-Pacific patients with NSCLC and 15% of Western patients (2). Although EGFR mutations occur more frequently in females, Asians, and never smokers, they are not restricted to patients with these characteristics. More than 50% of patients with EGFR mutations in the PIONEER study were not female non-smokers (2). These findings strongly support EGFR mutation testing in all patients with NSCLC and suggest that targeting EGFR for prevention will have a significant impact on controlling this disease.

Clinically approved therapeutics for NSCLC include small molecule tyrosine kinase inhibitors (TKIs) and monoclonal antibodies such as Cetuximab and Panitumumab (3–5). Advanced NSCLC patients harboring mutations in the EGFR gene show initially good responses to TKIs but ultimately relapse (6). The monoclonal antibodies are also limited in clinical use due to limited efficacy, toxicity, and the emergence of resistance (7, 8). While immune checkpoint blockade has shown encouraging effects in subtypes of NSCLC patients, relatively poor response in EGFR mutant NSCLC patients means that those patients who develop resistance to EGFR inhibitors have no effective treatment options. Thus, there is an urgent need to develop new strategies for safer and more efficacious therapeutics.

Key concepts in lung cancer disease control are prevention of lung cancer progression in patients with premalignant lesions and prevention of lung cancer recurrence in previously treated patients. Previous research indicates that EGFR mutations are not only an “early event” occurring during the initiation of lung cancer (9), but they are also the molecular driver of NSCLC (10, 11). The most common EGFR mutations (>90%) are exon 21 mutations resulting in L858R substitutions (~30–45%), and in-frame deletions in exon 19 (~40–60%). EGFR mutation-specific inhibitors have been developed for controlling NSCLC with EGFR mutations. However, second mutations (e.g., T790M) are a common mechanism of resistance. Therefore, alternative approaches to prevent tumor progression in EGFR mutated patients are needed.

Tumor-specific antigens (TSA) can be processed and presented by cell surface major histocompatibility complex (MHC) proteins as tumor mutation-encoding neoantigen (NeoAg) epitopes, which can serve as targets for tumor-specific T cell-mediated killing (12, 13). Several NeoAg vaccines have been developed from preclinical models with significant antitumor efficacy, and recent clinical trials using peptide-based or RNA-based NeoAg vaccine approaches have demonstrated successful induction of NeoAg-specific T cells response with improved clinical outcomes in melanoma patients (14–18). Furthermore, personalized NeoAg peptide vaccine trials for glioblastoma patients were shown to successfully induce antigen specific CD4+ and CD8+ T cell responses and increased T cell infiltration in tumors. Multiple epitopes specific to mutant EGFR were identified, including epitopes specific to L858R and T790M, and vaccines incorporating these epitopes induced immune responses, with clinical responses observed in some patients (19). These findings suggest that targeting neoantigens derived from EGFR mutations could be a useful treatment strategy for NSCLC patients with EGFR-TKI resistance. While the majority of these epitopes are MHC I specific, MHC II epitopes can be designed to target multiple HLA-DR alleles and are thus applicable for broader populations of cancer patients (20–24).

Induction of Th1 helper CD4+ T cell immunity is critical for immunomodulation-mediated cancer eradication (23). MHC II-restricted peptide vaccines can elicit NeoAg-specific Th1 immunity that orchestrates tumor-reactive CD8+ cytotoxic T lymphocyte (CTL) responses, for example, by increasing the number of antigens available to cytotoxic CD8+ T cells through the restoration of MHC class I expression on tumor and immune cells, providing cytokines to facilitate CD8+ responses, and by facilitating the generation of robust immunologic memory. Recently, Th1 cytotoxic T cell function has also been observed in various immune settings. Th1 Cytotoxic CD4+ T cells can directly kill cancer cells in an MHC II-restricted fashion through the secretion of granzyme B and perforin (12, 25, 26). We previously reported an MHC II-restricted EGFR multi-peptide vaccine that targets the wild-type EGFR protein and reduces EGFR-driven lung tumor burdens in a preclinical murine model (22). However, this vaccine is not specific to the mutant forms of EGFR; therefore, in this study, we extended our MHC II-restricted multi-peptide EGFR vaccine to include peptides for common EGFR mutations including the L746–S750 deletion, (Del19) T790M and L858R. We identified the most immunogenic epitopes specific to these mutations and tested this mutation-specific, multi-peptide vaccine, E^mut^ Vax, in both syngeneic and genetically engineered mouse models harboring mutant forms of EGFR.

## Material and methods

### Construction of lung cancer cell lines with L858R plasmids or/and T790M plasmids

For the syngeneic tumor model, we used the UN-SCC680 (UN680) lung cancer cell line that expresses the L858R/T790M or Del19 (L746-S750) mutant human EGFR protein. pBabe_EGFR(L858R/T790M) and pBabe_ EGFR(Del19) were gifts from Dr. Matthew Meyerson.

(Addgene plasmid # 32073 and #32062; http://n2t.net/addgene:32062; RRID : Addgene_32062 http://n2t.net/addgene:32073; RRID : Addgene_32073). Lentivirus particles were generated by co-transfecting pBabe_EGFR(L858R/T790M) and pBabe_EGFR(Del19) with packaging plasmids (pVSVG) into HEK293T cells. To overexpress EGFR L858R/T790M or Del19 mutations, UN680 cells were transduced with pBabe_EGFR(L858R/T790M) and pBabe_EGFR (Del19) lentivirus particles and then were selected in complete growth medium contains puromycin (1~2µg/ml). The protein expression of L858R/T790M, Del19, and phosphorylated EGFR was confirmed by western blot analysis.

### Mouse models and treatments

The mouse NSCLC UN680 cells derived from the A/J background were a generous gift from Dr. Jackeline Agorreta. UN680-derivatives UN680^L858R/T790M^ and UN680^Del746-750^ were established to represent NSCLC harboring common EGFR mutations. Five- to six-week-old female A/J mice were purchased from the Jackson Laboratory. After one week, they were grouped according to similar body weights into two groups: (1) adjuvant control mice receiving CFA/IFA; (2) E^mut^ Vax group receiving EGFR mutant-specific peptides suspended in adjuvant once per week for a total of four doses. For the syngraft model, UN680^L858R/T790M^ or UN680^Del746-750^ cells (5 × 10^6^ cells in 0.1 ml of PBS) were inoculated subcutaneously into the flanks of experimental and control mice one week after the fourth vaccine (Day 0). The growth of UN680^L858R/T790M^ or UN680^Del746-750^ tumors was measured every four to six days by a digital caliper. Tumor volumes were calculated using the formula: V = (length × width × width)/2 (27).

To further test E^mut^ Vax in the prophylaxis setting, an inducible genetically engineered mouse model was used. Transgenic mice harboring human EGFR containing both L858R and T790M mutations (EGFR^L858R/T790M^ mice) on a C57BL/6 background were requested from the NCI mouse repository. EGFR^L858R/T790M^ mice were bred with CCSP mice that expresses the Tet-on Clara Cell Secreted Protein (CCSP) on the C57BL/6 background to generated double transgenic mice (CCSP-EGFR^L858R/T790M^), which will have lung-specific expression of the transgene EGFR^L858R/T790M^ upon doxycycline induction. All mice were housed in the Biomedical Resource Center at the Medical College of Wisconsin, Milwaukee, WI. All procedures were approved by the institutional animal care and use committee (IACUC). For primary lung cancer experiments, 4-5 week-old CCSP-EGFR^L858R/T790M^ mice were divided into the following two groups: (1) adjuvant control mice that received complete Freund’s adjuvant (CFA) and incomplete Freund’s adjuvant (IFA); (2) E^mut^ Vax treated mice that received the EGFR mutants specific peptides suspended in adjuvant. Doxycycline-containing diets (TD. 01306, Envigo Teklad) were administered as food pellets starting one week after initial 4-weekly vaccinations.

### Neo-epitope prediction

To predict HLA class II epitopes for each mutant EGFR of interest in our study, we used a newly developed algorithm, MixMHC2pred (28). Briefly, each mutation was represented by a sequence that was centered by the mutation site with 14 amino acids extended from both sides, such that the typical sequence length is 29 amino acids (14 + mutation site + 14). We then took 15-amino-acid sliding windows along these sequences and generate an average of 9 different peptides for each mutation, then ran each peptide (mutant form or wild type form) with the most common human HLA alleles to generate a MixMHC2pred score, % Rank_best, which corresponds to a percentile rank (best score is about 0, worst score is 100) of the given peptide expected to be presented by the alleles.

### Vaccine preparation and immunization

**Figure 1:**
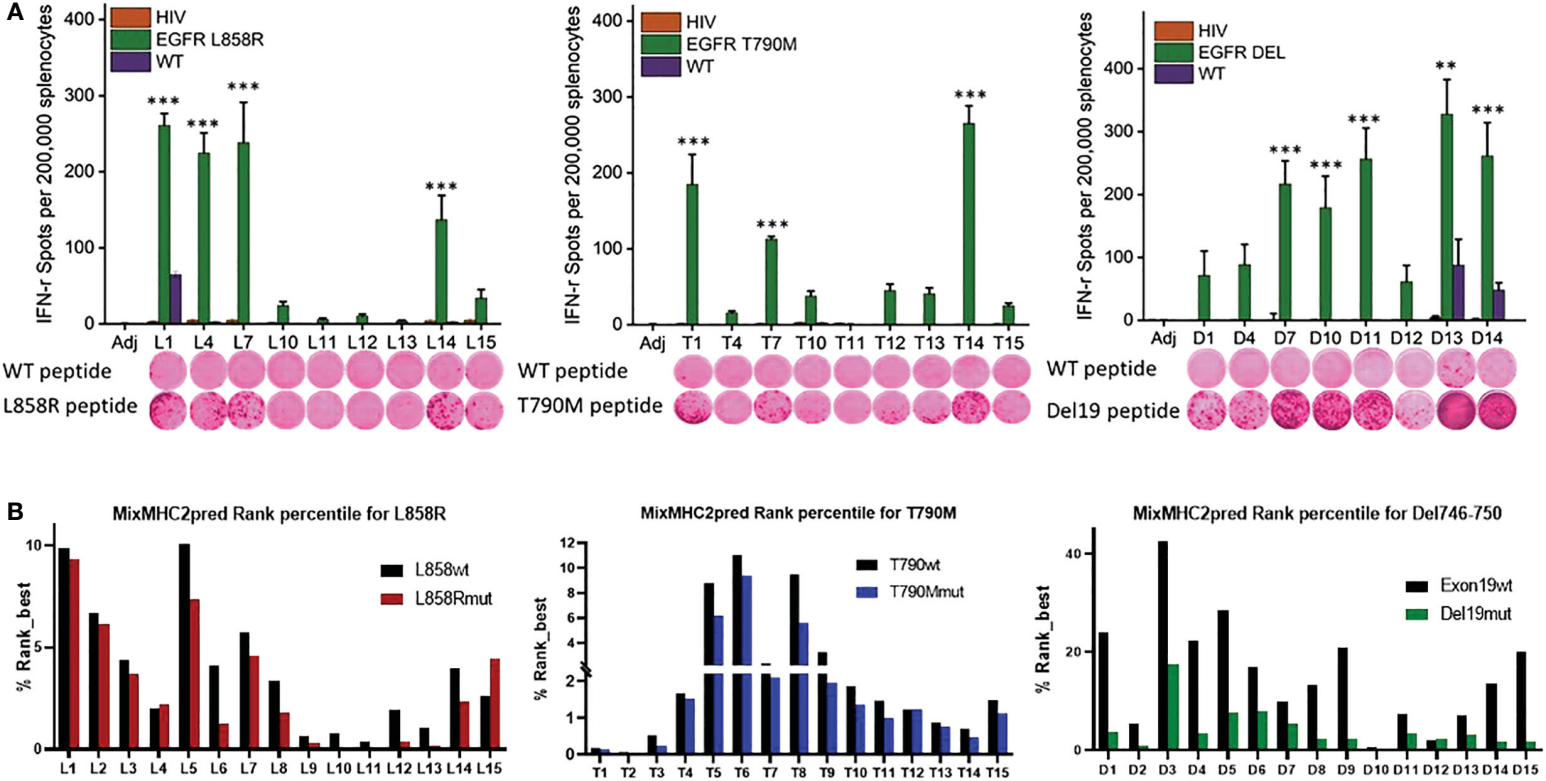
Identification of immunogenic epitopes specific to common EGFR mutations. **(A)** Quantified (bars in panels) and representative (inset images) ELISPOT results showing T cell responses from naïve mouse splenocytes stimulated with wild-type peptides (first row images) or mutation-specific peptides (second row images). Mice were vaccinated 4 times weekly with individual E^mut^ peptides, splenocytes were collected 7 days after the last vaccination, and the splenocytes pulsed with negative HIV control peptide, each EGFR wild type or mutant peptide. After 72h of incubation, ELISPOT assays were performed, plates were scanned, and spot numbers were statistically analyzed. Data are shown as the mean ± SE of three replicate wells per group, n=5, *P ≤ 0.05, **P ≤ 0.01, ***P<0.001. **(B)** MixMHC2pred prediction for best-ranked peptides specific to L858R (top panel), T790M (middle panel) and Del19 (bottom panel) EGFR mutations, the general score (%Rank_best) was predicted with the default algorithm setting that includes over 50 most common human MHCII molecules, and the score represents the percentile rank of the peptide expected to be presented and immunogenic with these common alleles.

**Figure 2:**
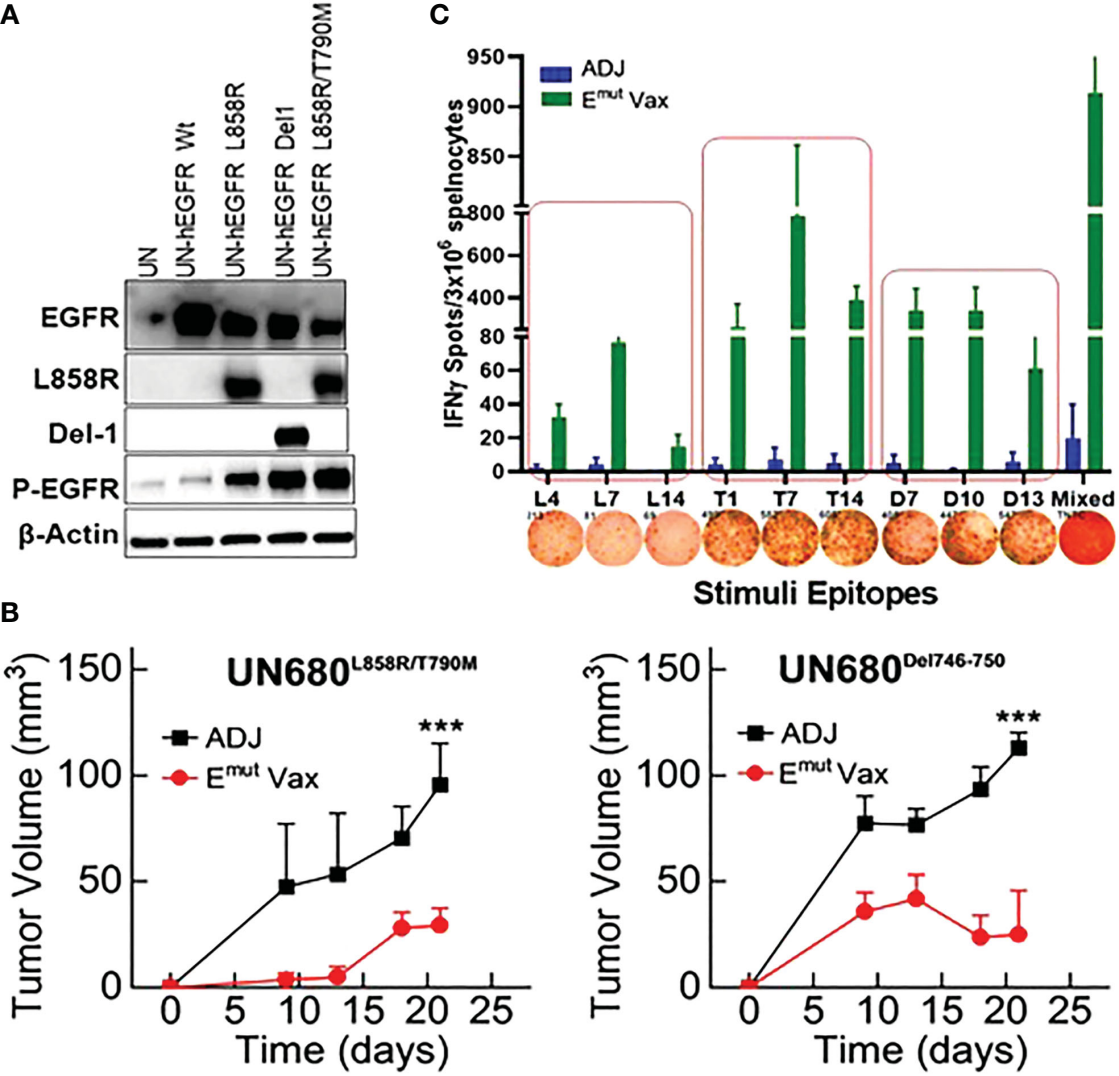
E^mut^ Vax inhibits lung tumor progression in syngraft models. **(A)** Western blots to confirm expression of different mutant forms of EGFR in UN680 cells. **(B)** Tumor volume quantitation over time in the L858R/T790M resistant UN680 syngraft tumor model (left panel) or UN680 Del19 syngraft model (right panel). **(C)** Quantified and representative (insets) ELISPOT results from the spleens harvested at the endpoint of the efficacy study from the UN680 syngraft models. Data were combined from both L858R/T790M resistant or Del19 syngraft models. Data are the mean ± SE, n=10, ***P ≤ 0.001 vs Adj Ctrl.

EGFR mutation-specific peptides (**Supplementary Table 1**) that are 100% identical between humans and mice were synthesized with >95% purity by Genemeds, and their identity was confirmed by HPLC. Peptides were dissolved in 10% DMSO/PBS at 10mg/ml. CFA and IFA were used as adjuvants throughout the study. The vaccine was prepared by mixing an equal volume of peptides in PBS with CFA or IFA till a uniform water-in-oil emulsion formed. Each mouse received 50 μg of each peptide for one vaccine injection. The total vaccine volume was 100 μL/mouse. Mice were injected subcutaneously in the right flank with the first vaccination prepared in CFA starting at 5 weeks of age, followed by 4 weekly booster vaccines prepared in IFA, and then additional boosters administered every 4 weeks thereafter in the GEMM as shown in **Figures 1A**, **2A**.

### ELISpot assay

Spleens were mechanically mashed through a 40 μm cell strainer (BD). Red blood cells were lysed in ACK lysis buffer for 2 min, neutralized with complete RPMI1640 medium, centrifuged at 1000*g* for 3 min, then re-suspended in T cell medium (RPMI1640 plus 10% FBS, 1% pen/strep and 0.05 mM β-ME). 100 µl of splenocytes (3.0x10^5^ cells/ml) were seeded onto a MAIPS4510 Multiscreen 96-well plate that was coated with anti-IFN-γ capture antibody. The cells were stimulated with mutation-specific peptides, concanavalin A (positive control), an HIV peptide (negative control), and wild-type peptides (negative control) for 72hr. The plates were then washed four times with PBS with 0.5% Tween-20 and the anti-IFN-γ detection antibody (BD) was added for overnight culture at 4°C. After washing with PBS four times, HRP-streptavidin was added for 1hr incubation, and then developed by adding AEC substrate following the manufacturer’s instruction. Plates were scanned and quantified by an automated plate reader system (CTL Technologies).

### Cytokine analysis

Splenocytes isolated from ADJ or E^mut^ Vax group of mice were stimulated with Emut peptides for 72 hr, then the supernatant was harvested and sent to EVE’s Technologies (Calgary, AB Canada) for analysis with Mouse Cytokine Array (MD31, https://www.evetechnologies.com/product/mouse-cytokine-array-chemokine-array-31-plex/).

### Magnetic resonance imaging

To monitor lung tumor development, CCSP-EGFR^L858R/T790M^ mice were imaged by a 9.4T MRI (Bruker, Billerica, MA) equipped with a customized birdcage style quadrature coil. Animals were anesthetized with isoflurane (2% for induction and ~1.5% for maintenance). Mouse body temperature, heart rate, and respiratory rate were continuously monitored throughout the imaging procedure. Both respiratory and cardiac gating using an electrocardiogram was used to ensure that images were acquired during latent periods of the respiratory cycle and at a consistent point during the cardiac cycle. Tumors were scanned using a multi-slice, multi-echo acquisition (MSME). The following parameters were used to acquire the images: TE = 8.07 ms, TR ≥ 400 ms (variable), matrix = 128 × 128, on average, 20 axial slices. The Dicom viewer was used to process the coronal slice images.

### H&E staining for lung tumor counting

Mouse lungs were inflated and formalin-fixed overnight and transferred to 70% ethanol. Fixed lung tissues were then paraffin-embedded (Sakura Tissue Tek VIP5), sectioned at 5 μm for H&E staining. The NanoZoomer slide scanner was used to scan and measure.

H&E slides of lungs were also scored according to a grading scheme that was previously developed for this same GEMM (29). Briefly, 0 indicates no noticeable lesions; 1 indicates minor abnormalities, the majority of the lung is intact with rare tiny atypical adenomas and multifocal mild atypical type II pneumocyte hyperplasia; 2 indicates minor lesions: small areas of mild atypical hyperplasia or atypical adenomas, large regions are unaffected; 3 indicates moderate lesions: large regions of atypical hyperplasia; occasional well differentiated and demarcated adenocarcinomas with no lymphovascular invasion; 4 indicates moderately severe lesions: multifocal to coalescing adenocarcinomas and atypical adenomas that affect the majority of a lung lobe; 5 is for severe lesions: diffuse involvement, adenocarcinoma is invasive with lymph vascular invasion.

### Flow cytometry

To analyze the tumor-infiltrating T cells, tumors were first minced into 1-2 mm^3^ pieces and then processed into single cell suspensions using mouse tumor dissociation kits (Miltenyi Biotec, CA) according to the manufacturer’s instructions. Tumor-infiltrating leukocytes (TILs) were enriched by centrifugation on 40–70% Percoll (GE) gradients. Cells were first stained with fixable live/dead dye in PBS, after wash then followed with surface staining buffer containing fluorochrome-conjugated anti-CD45, anti-CD4, anti-CD8a, anti-CD44, and anti-CD62L antibodies. For intracellular cytokine staining (ICS), cells were stimulated for 4 h in T medium (RPMI1640, 10% FBS, 2 mM L-glutamine, 50 μM 2-mercaptoethanol, 1% penicillin-streptavidin) containing KRas peptides (10ug/ml), 1x monensin, 1x Brefeldin A (Thermofisher Sci). Stimulated cells were then stained with fixable live/dead dye in PBS, followed by cell surface staining buffer containing anti-CD45, anti-CD4, and anti-CD8 antibodies. After washing, the cells were fixed with 2% paraformaldehyde at room temperature for 10min, permeabilized with FOXP3 Fix/Perm Buffer Set (Biolegend), and stained with 1x Perm buffer containing anti-granzyme B, anti-IFN-γ and anti-TNFα antibody, and analyzed by flow cytometry. T cells stained with isotype control antibody were used as negative controls. Flow cytometry was analyzed using an LSR-II flow cytometer (Becton Dickinson). Data were processed with FlowJo software (Tree Star).

### scRNA-seq data analysis

Lungs from transgenic CCSP-EGFR^L858R/T790M^ mice were perfused with PBS through the right ventricle letting the blood flow out of the premade incision at the left ventricle till the lungs turned white, then tumors were microdissected from each mouse in the adjuvant or E^mut^ Vax group at the end of the efficacy study. scRNA-seq and data analysis was conducted as previously reported (30).

### Statistical analysis

GraphPad Prism 9.0 software was used for statistical analysis.

Two-tailed Student’s t-test or one-way ANOVA were used for multiple columns or group analyses. *P< 0.05, **P< 0.01, or ***P< 0.001 were considered as significant.

## Results

### Identification of immunogenic epitopes in specific EGFR mutations

To identify the most immunogenic epitopes, each somatic mutation was centered by a sequence with 14 amino acids upstream and downstream of the mutation site, such that the sequence length was 29 amino acids. 15-amino-acid sliding windows along these sequences were taken (**Supplementary Table 1**) and every single peptide was evaluated in naïve A/J wild-type mice. Mice were first given 4 weekly vaccinations with each different single epitope, and splenocytes were harvested 7 days after the last vaccination for testing in IFN-γ ELISPOT assays. Splenocytes were re-stimulated with either the vaccinated mutant forms of epitopes, the corresponding wildtype peptides, or a nonspecific HIV peptide. As shown in **Figure 1A**, L858R peptides L1, L4, L7 and L14 induced strong immune responses specific to the mutant forms of the vaccine peptides. In contrast, all vaccinated mice showed no response to nonspecific HIV epitope stimulation. Of note, L1 elicited a relatively weak response to the wild-type form of the EGFR peptide, implying L1 vaccination may lead to potential side effects by targeting wildtype EGFR. For the T790M mutation, T1, T7, and T14 exhibited strong immune responses to the specific mutant forms of the peptides; none of these peptides induced immune recognition of the wildtype EGFR or nonspecific HIV peptides. For Del746-750, almost all the mutant epitopes generated a strong immune response except for D1, D4 and D12 in naïve mice, and these immune responses were specific to the mutant epitopes but not to nonspecific HIV or wildtype EGFR epitopes, except for D13 and D14, which induced weak responses to the wildtype EGFR (**Figure 1A** **)**.

Using the MHC II prediction algorithm MixMHC2pred, which predicts MHC II binding, epitope processing, cleavage, and antigen presentation, we compared the MixMHC2pred score between wildtype and mutated epitopes with the most common HLA-II alleles in humans. The MixMHC2pred general score (%Rank_best) represents the percentile rank of the peptide expected to be presented and immunogenic with these common alleles, the best score is about 0, and the worst score is 100, the lower score represents a better predicted immunogenicity. The mutations of EGFR shifted these epitopes towards a more immunogenic state. As shown in **Figure 1B** lower panel, Del19mut epitopes had lower %Rank_Best scores from this algorithm versus wildtype epitopes, which indicates better binding and presentation and is in line with our findings that the majority of the Del746-750 epitopes demonstrated strong and specific immune responses when tested in naïve mice. Mice immunized with adjuvant didn’t develop antigen-specific IFN-γ response (*P*<0.001, **Figure 1A**). To include peptides specific against mutant forms of EGFR, peptides L4, L7 and L14 (**Figure 1A** upper left panel), T1, T7, T14 (**Figure 1A** lower panel), and D7, D10 and D13 (**Figure 1A** upper right panel) were chosen to formulate a multi-peptide E^mut^ Vax in the preventive efficacy study.

### E^mut^ Vax inhibits lung tumor formation in syngeneic tumor models

To test the preventive efficacy of the newly formulated E^mut^ Vax in immunocompetent NSCLC mouse models, we first used a syngeneic lung tumor, UN680, a mouse NSCLC cell line developed in the A/J mouse background after chronic chemical carcinogenesis, which is responsive to multiple immunomodulatory agents including anti-PD1 (31). The UN680 cells were engineered to overexpress either the L858R/T790M double mutation or Del746-750 mutation, and expression of the EGFR mutant forms was confirmed by western blotting (**Figure 2A**). The mutant lines also express higher levels of phosphorylated EGFR (data not shown). In this model, E^mut^ Vax was given subcutaneously weekly for four weeks, and the tumor cells were inoculated one week later. Compared to the adjuvant control group, E^mut^ Vax decreased tumor load by ~70% (**Figure 2B**) for both EGFR mutant tumor cell lines, indicating a strong protective effect. IFN-γ ELISpot assay of splenocytes showed that vaccinated animals significantly expressed IFN-γ T cell response upon peptide re-stimulation (**Figure 2C**). The reactivity to the EGFR peptides was highly specific to this mutant EGFR, which indicated by the lack of response present in non-vaccinated but tumor-bearing animals, suggesting that the immune responses were induced by vaccination but not the boosting effect promoted by tumor inoculation. Although the peptides chosen for the E^mut^ Vax were all immunogenic when tested alone in naïve mice, when combined into one vaccine, the ELISPOT immune response was strongest with T790M and Del746-750 specific peptides.

**Figure 3:**
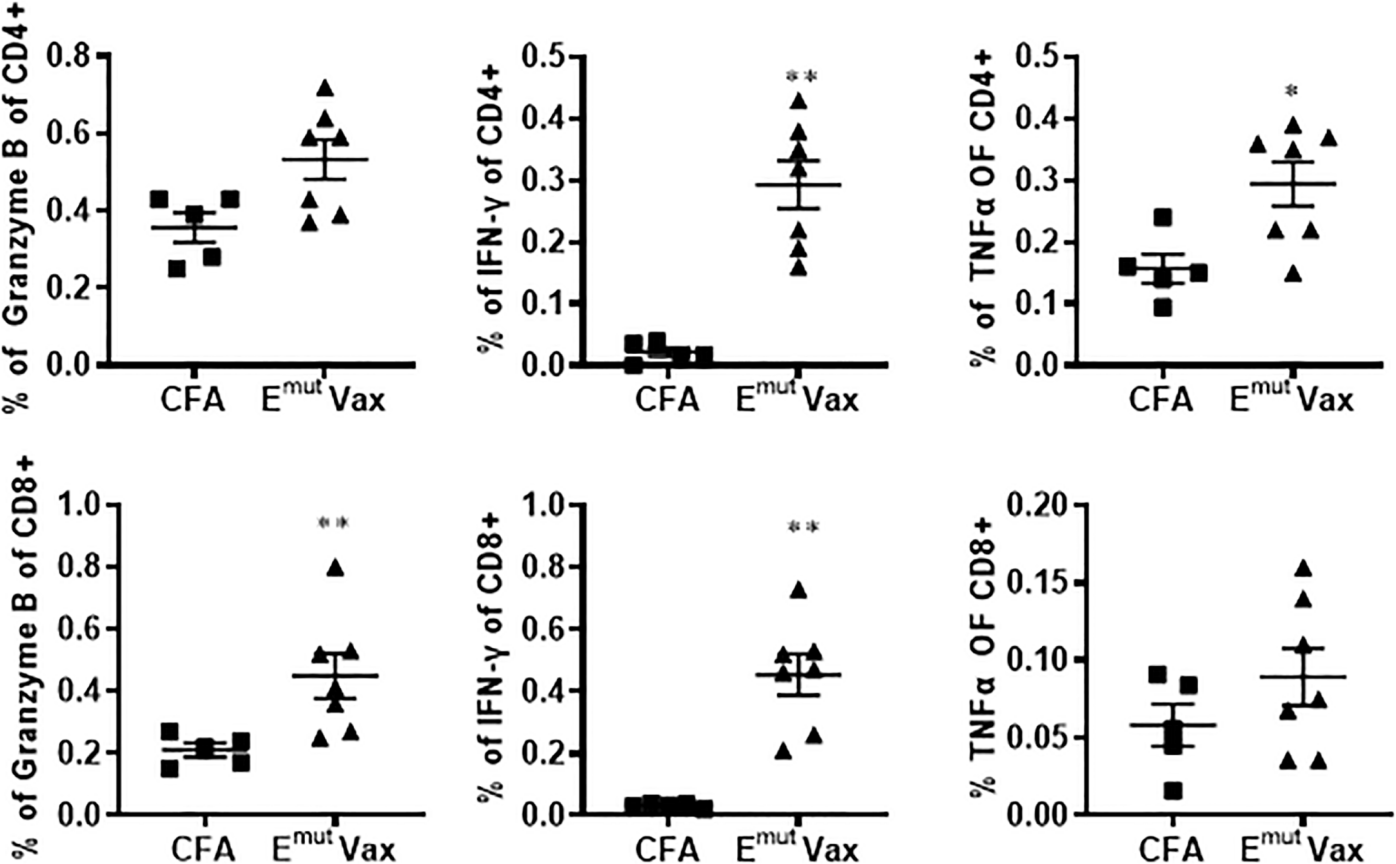
E^mut^ Vax increased Th1 immune responses in a tumor-bearing syngraft model. Spleens were harvested from mice in the UN680 Del19 syngraft model, restimulated with E^mut^ multi-peptides, and frequencies of Th1 cytokine and cytolytic granule-producing CD4+ and CD8+ were analyzed. Data are shown as the mean ± SE of three replicate samples per group, n=5 in ADJ and n=7 in E^mut^ VAX, *P ≤ 0.05, **P ≤ 0.01 vs ADJ Ctrl.

Flow cytometry analysis from splenocytes harvested at the endpoint revealed that overall percentages of CD4+ and CD8+ cells of vaccinated animals were not significantly different from adjuvant control groups. However, the frequencies of IFN-γ-secreting CD4+ and CD8+ T cells was significantly increased in splenocytes harvested from the vaccinated mice (**Figure 3**). In the meantime, the frequencies of CD4+ T cells secreting granzyme B and CD8+ T cells secreting TNF-α were also showed a trend of increase (**Figure 3**). According to these findings, Emut Vax-induced T cell responses are specific to the mutant peptides and aid in the development of an adaptive anti-tumor response. Since none of the animals responded to an irrelevant antigen (HIV peptides), this further confirming the idea that the immune response seen in the vaccinated group was highly specific to mutant EGFR.

### E^mut^ Vax prevents lung tumorigenesis in the EGFRL858R/T790M genetically engineered mouse model

**Figure 4:**
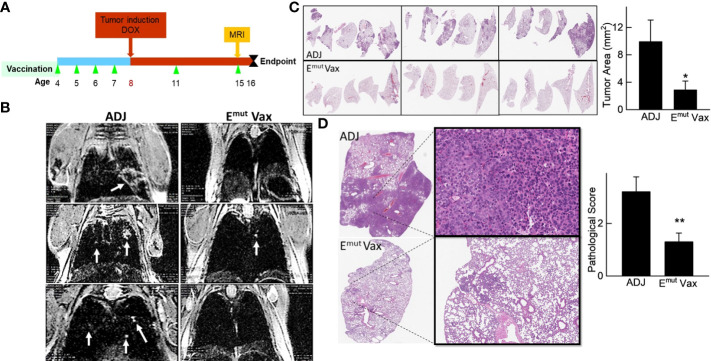
E^mut^ Vax inhibits lung tumor progression in a genetically engineered mouse model. **(A)** Experimental design outlining the timing of vaccine administration and tumor induction with DOX diet, the time point for MRI monitoring, and the experimental endpoint. **(B)** Representative MRI images from mice injected with either adjuvant control or E^mut^ Vax. **(C)** Representative lung lobes (left panel) and quantification of tumors (right panel) from each group. **(D)** Representative lung lobes (left panel) and pathological scores (right panel) from each group. Data are shown as the mean ± SE, n=10, *P ≤ 0.05, **P≤0.01.

We employed mice designed to express full-length human EGFR with the L858R and T790M mutations to assess Emut Vax. This animal model can develop lung adenocarcinomas quickly, are resistant to TKI treatment, and represent patients who have undergone first line TKI standard of care therapy with no further treatment options. Mice were vaccinated according to the treatment schedule in **Figure 4A**, and doxycycline administration was started one week following the last vaccine. Representative MRI imaging showed that while the non-vaccinated animals receiving adjuvant treatment displayed hypercellular lung parenchyma with few air spaces, the vaccine group displayed normal alveolar structures with little evidence of lung disease but some evidence of aggregates with a lymphoid character (**Figure 4B**).

As previously reported and outlined in the Methods section, tumors in the transgenic animals were located within the lung parenchyma, these tumors were hard to be quantified on the surface of the lung. Slides from different anatomic regions were analyzed. The tumor load within each animal was averaged to evaluate changes. Vaccination decreased tumor burden after induction of the EGFR transgene. Vaccinated animals demonstrated an average of 2.5 mm^2^ tumor area, versus an average of 10.76 mm^2^ tumor area found in unvaccinated animals, equating to a 75% reduction in tumor burden (**Figure 4C**; P = 0.0014**)**. To quantify the histopathology response to E^mut^, the morphology and extent in the tumors were graded, with scores ranging from 0 (no lesions) to 5 (multifocal to consolidating adenocarcinoma) based on previously described criteria (29). The histopathologic severity grades were mostly 4 and up in the adjuvant group, and the lung lobes were affected with moderately severe lesions, consisting of multifocal to coalescing adenocarcinomas, atypical adenomas and adenocarcinomas. E^mut^ Vax prevented tumor progression and decreased pathological score from 3.2 in the adjuvant group to 1.3 (**Figure 4D** **)**; most tumors were grade 2, which is defined as occasional atypical adenomas with rare borderline/early adenocarcinoma.

### Single-cell gene expression landscape generated from GEM model tumor samples

**Figure 5:**
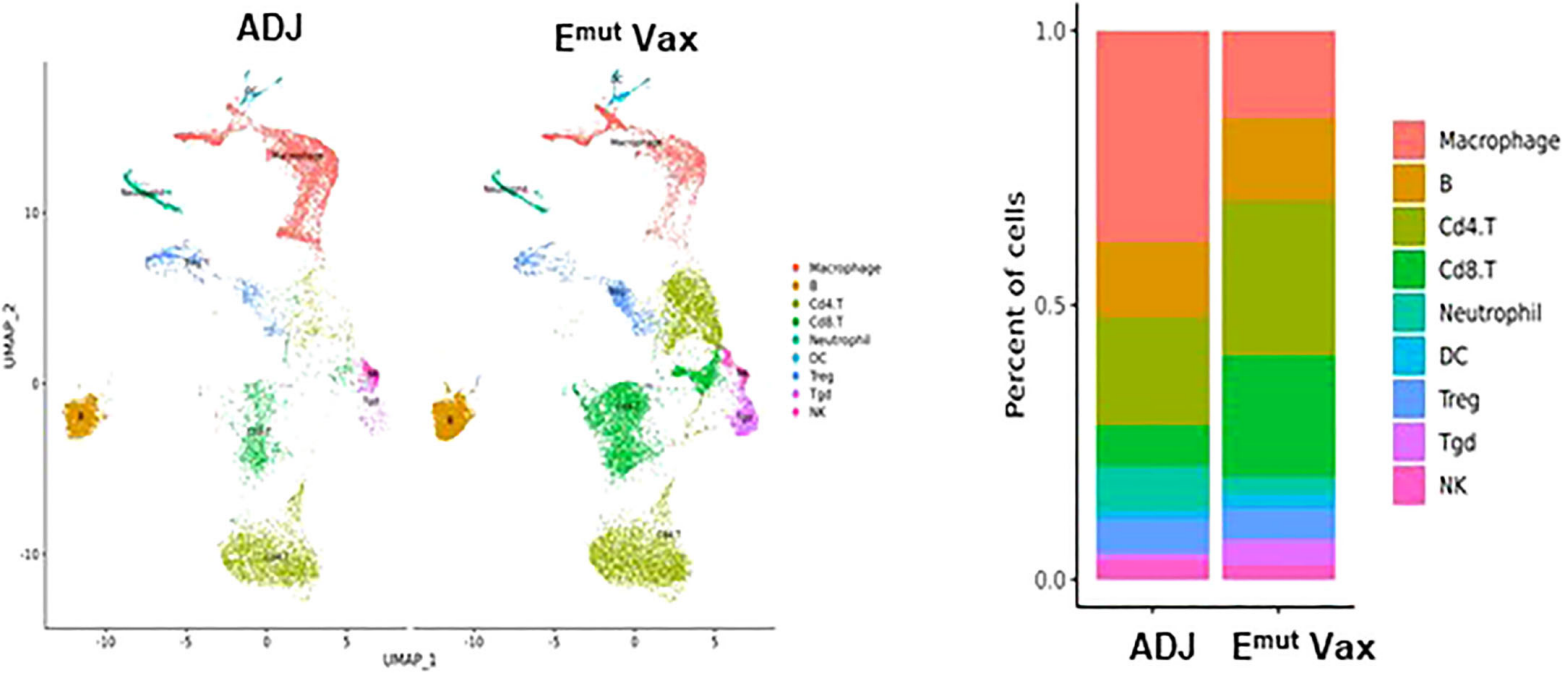
Clustering analysis of scRNA-seq data from tumor-infiltrating cells in adjuvant controls and E^mut^ Vax treated mice. Left panel: The overall immune landscape across adjuvant control and E^mut^ Vax treatment groups. Right panel: The percentage changes of each immune cell population between the adjuvant control and E^mut^ Vax treatment groups. E^mut^ Vax versus ADJ group cell percentage comparison: Macrophage: P = 0.008; B cells: P = 0.09; Cd4+ T cells: P = 0.02; Cd8+ T cells: P = 0.03; Neutrophil: P = 0.05; DC: P = 0.043; Treg: P = 0.11; Tgd: P = 0.052; NK: P = 0.36.

To reveal the specific changes in tumor infiltrating lymphocyte compositions and gene expression in E^mut^ VAX treated animals, scRNA-seq was performed on lung tumors harvested from transgenic CCSP-EGFR^L858R/T790M^ mice in the adjuvant or E^mut^ Vax group at the end of the efficacy study. We utilized the Seurat package (32, 33) to perform fine clustering of RNA sequenced single cells in mouse lung tumor samples from the adjuvant and E^mut^ VAX treatment groups. Single-cell gene expression data were aligned and projected in 2 dimensions through UMAP (34). The gene expression patterns of canonical markers were analyzed to characterize different immune cell populations in the tumor samples. Nine different immune cell populations were detected in the lung tumor samples including CD4+ T cells, Treg, Tgd (gamma/delta T cells), CD8+ T cells, neutrophils, B cells, natural killer (NK) cells, macrophages, and dendritic cells (DC), etc. (**Supplementary Figures 1A, B** **)**. Compared to the adjuvant control group, E^mut^ VAX treatment increased CD4+ T, CD8+ T, Tgd cells, B cells, and DC, and decreased macrophages, neutrophils, and Treg cells (**Figure 5** **)**. These changes suggest that overall compositional changes in immune cells towards those exhibiting anti-tumor reactivity occur after E^mut^ VAX treatment.

### EGFR vaccination increased the proportions of Th CD4+ T cells

**Figure 6:**
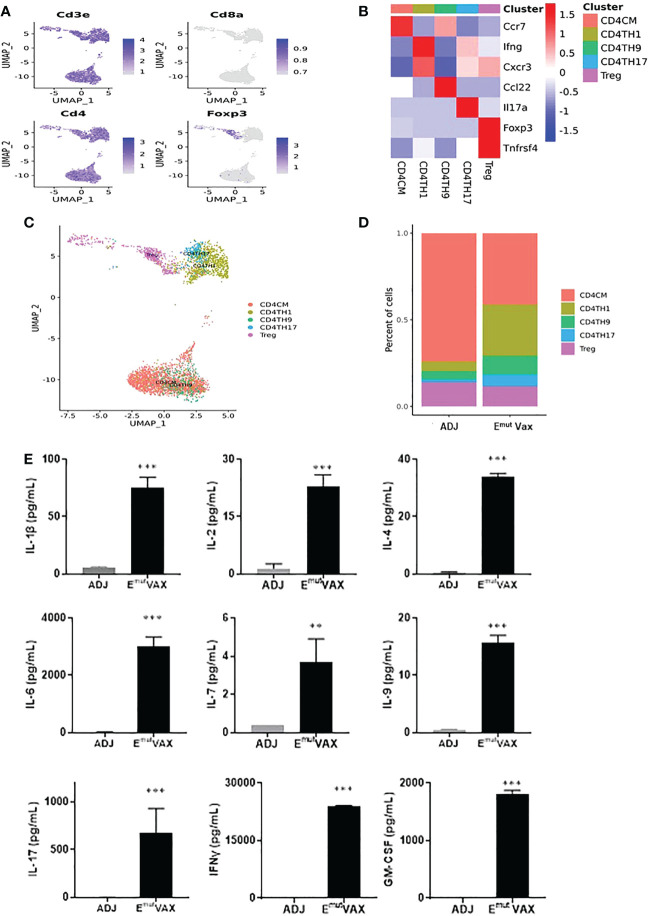
E^mut^ Vax treatment stimulated anti-tumor CD4^+^ effector T cells within tumor-bearing lungs. **(A)** Canonical CD4^+^ T cell marker expression based on scRNA-seq data. **(B)** Heatmap of the expression markers for each CD4^+^ T cell subset. **(C)** The distribution of each CD4^+^ T cell subset. **(D)** Percent changes of the CD4^+^ T cell subsets across control and E^mut^ Vax treatment groups. Data are shown as the mean ± SE, n=5, CD4CM: P = 0.032; CD4TH1: P = 0.016; CD4TH9: P = 0.037; CD4TH17: P = 0.046; Treg: P = 0.12. **(E)** Cytokine analysis from E^mut^ Vax treated mice vs ADJ mice. Splenocytes were harvested at the endpoint, restimulated with E^mut^ peptide vaccines for 72hr, then supernatant was collected for the cytokine analysis. Data are shown as the mean ± SE, n=5, **P ≤ 0.01, ***P ≤ 0.001 vs ADJ control.

We analyzed all CD4+ T cells in mouse lung tumors from the control and treatment groups (**Figure 6A**) and identified five types of CD4+ T cell subsets, i.e., CD4CM (CD4+ central-memory T cells), CD4TH1 (Th1 cells), CD4TH9 (Th9 cells), CD4TH17 (Th17 cells) and Treg cell subpopulations (**Figures 6B, C** **)**. E^mut^ Vax treatment significantly increased proportions of anti-tumor CD4+ Th1, Th9 and Th17 cells in the lung tumors, while the vaccine decreased the abundance of Treg cells (**Figure 6D**). Cytokine analysis from Emut Vax treated spleens also confirmed the upregulated IFN-Ɣ secretion, as well as IL-1β, IL-2, IL-4, IL-9 and IL-17 upregulation (**Figure 6E**). IL-1β together with IL-4 could induce antitumor Th9 cell differentiation even in the absence of TGFβ. IL-2 signaling is important for the development of both Tregs and Th9 cells, while the enhanced IL-6 is critical for the inhibition of Foxp3+ Tregs and favors Th17 differentiation. Our results indicate that the anti-tumor function of the CD4+ T cells, especially CD4+ Th1 cells, was significantly enhanced by Emut Vax, together with the complex cytokine network that may control the transdifferentiation of different T helper lineages.

### EGFR vaccination increased the proportions of cytotoxic CD8+ T cells

**Figure 7:**
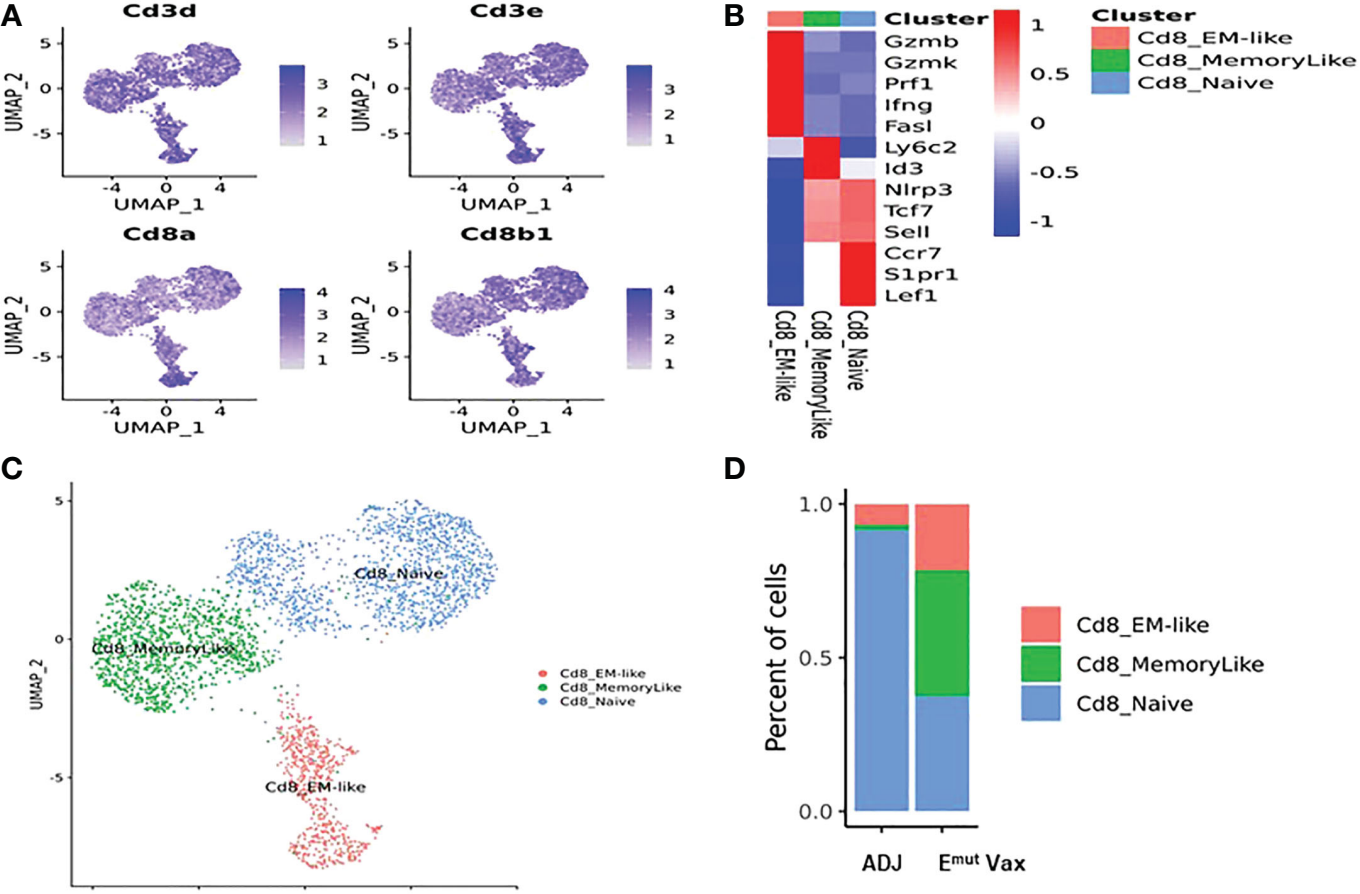
E^mut^ Vax treatment increased anti-tumor CD8^+^ cytotoxic T cells within tumor-bearing lungs. **(A)** Canonical CD8+ T cell marker expression based on scRNA-seq data. **(B)** Heatmap of the expression markers for each CD8^+^ T cell subset. **(C)** Distribution of each CD8^+^ T cell subset; **(D)** Percent changes of the CD8^+^ T cells subsets across adjuvant control and E^mut^ Vax treatment groups. CD8_EM-like: P = 0.013; CD8_MemoryLike: P = 0.007; CD8_Naive: P = 0.009.

To determine the effects of E^mut^ Vax treatment on different subsets of CD8+ T cells in mouse lung tumors, deep clustering of the CD8+ T cells was performed. Identification of CD8+ T cell subsets was performed by using canonical markers (**Figure 7A**). The TILPRED program was used for unsupervised clustering of CD8+ T cells (35) and three CD8+ T cells subsets with distinct transcriptomic profiles including effector memory (EM)-like, memory-like, and naïve cells were identified (**Figures 7B, C** **)**. In the lung tumor microenvironment, E^mut^ Vax treatment greatly increased the abundance of the anti-tumor EM-like CD8+ T cells (**Figure 7D**). The frequency of memory-like CD8+ T cells was also increased, while that of naive CD8+ T cells was decreased by E^mut^ Vax treatment. These results suggest that the E^mut^ Vax improves the overall composition of antitumor CD8+ T cells in the lung tumor microenvironment.

We further analyzed the other immune cell populations in the mice lung TME for their association with the E^mut^ VAX treatment. Macrophages were subdivided into the M1 and M2 macrophages according to the marker gene expression (**Supplementary Figures 1B**, **2**). It was found that the abundance of the anti-tumor M1 macrophages increased and the pro-tumor M2 macrophages decreased greatly in the E^mut^ VAX treatment groups compared to the control group (**Supplementary Figure 2D**). These indicated that the anti-tumor response by E^mut^ VAX treatment may also be mediated *via* macrophages. We also identified two subpopulations of the NK cells: the CD27-CD11b+ NK cells corresponding to the human CD56dim NK cells which have strong cytotoxic activity and the CD27+CD11b- NK cells corresponding to the human CD56bright NK cells which are pre-mature and less cytotoxic (8). The total NK cells were identified by the expression of the Ncr1 gene (**Supplementary Figure 3A**) and the respective signature scores were used to locate each of these two NK cell subtypes (**Supplementary Figures 3B, 3C**). Under the E^mut^ VAX treatment, the proportion of the cytotoxic CD27-CD11b+ NK cells increased significantly while that of the pre-mature CD27+CD11b- NK cells decreased (**Supplementary Figure 3D**), suggesting the involvement of the cytotoxic NK cells in the anti-tumor function of the E^mut^ VAX.

## Discussion

EGFR is one of the most frequently mutated oncogene families that drives human NSCLC development (23). EGFR mutations are not only an “early event” occurring during the initiation of lung cancer (9), they are the molecular driver of NSCLC (10, 11). Therefore, approaches to target EGFR mutation to prevent tumor progression in EGFR mutated populations are an urgent need. Several EGFR inhibitors have been demonstrated to be highly effective in cancer patients with EGFR mutants. However, these inhibitors mostly do not show a significant benefit on overall survival due to secondary mutations that result in resistance to TKIs (18). Few strategies are available once patients become resistant to TKI treatments.

Deletions in exon 19 OR a point mutation in exon 21 L858R are the most common EGFR alterations associated with TKI sensitivity. About 90% of all EGFR alterations are these two alterations (36), which can serve as ideal targets for cancer immunoprevention. Here, we provided the first proof of principle study showing that a multi-peptide vaccine specific to common EGFR mutations (E^mut^ Vax) elicits a strong immune response and provides protection against lung tumorigenesis when given in the preventive setting. We selected peptides that induced a relatively strong Th1 response for the final vaccine formulation. E^mut^ Vax was very effective to prevent tumor development, which showed a 75% decrease in tumor volume in a highly aggressive transgenic model that recapitulates salient features of human EGFR lung cancers. The amino acid peptides we used are 100% identical between humans and mice. Furthermore, we used the newly developed MixMHC2 to predict the most common human HLA class II proteins and found the mutated epitopes generated better MHC-II binding and presentation for human HLA class II. Therefore, the peptides formulated E^mut^ Vax not only had strong immunogenicity in mice but also have a high likelihood of human class II binding, making it possible to be directly translated to clinical studies. EGFR mutant-specific immune responses were not seen in diseased, non-vaccinated adjuvant control mice, indicating that the mice are tolerant to the transgene product and that prophylactic vaccination with E^mut^ Vax can break this immune tolerance resulting in inhibition of tumor development.

Therapeutic cancer peptide vaccines have shown limited efficacy in clinical trials, most likely due to the profound immune suppression that develops in subjects with advanced tumors (37). The role of peptide vaccines has not been extensively studied as prevention prior to the establishment of tumor-induced immune suppression. Personalized vaccines have also been developed against multiple neo-Ags, however, most have focused on specific MHC I epitopes (12, 14, 38, 39). MHC II epitopes can be designed to bind to multiple HLA-DR alleles and are thus applicable to broader populations of at-risk or cancer patients. We tested a multi-peptide EGFR vaccine against multiple mutant epitopes in an EGFR mutant transgenic lung adenocarcinoma mouse model. The prophylactic administration of this multi-valent vaccine can prevent tumorigenesis driven by mutant EGFR. The high incidence of EGFR mutant tumors in the East Asian population supports the concept of performing a true prevention trial with this vaccine. Because our vaccine is directed against specific mutations, vaccination may be a powerful adjuvant treatment to prevent the spread of treatment-resistant disease. The low levels of immune suppressive tumor microenvironment mediated by EGFR inhibitor treatment may create a window of opportunity, in which vaccination can more effectively generate anti-tumor immunity for secondary prevention.

In the current study, E^mut^ Vax predominantly elicited a Th1 T cell response without significant induction of a Th2 response. Consistent with our previous findings, E^mut^ Vax significantly increased tumor infiltrating CD4+ T cells. Interestingly, vaccination also markedly enhanced tumor infiltrating CD8+ T cells and boosted both the central memory (T_CM_) and effector memory (T_EM_) CD8+ T cell frequencies and function in CD4+ and CD8+ T cell subsets. Both T_CM_ and T_EM_ are antigen-experienced T cells, with T_EM_ functions as sentinels for immediate protection, while T_CM_ represents long term memory population that provide protection from a systemic challenge and can generate a second wave of effector cells. The enhanced TEM and TCM together might confer the profound host protection during tumorigenesis. The proliferation and recruitment of CD8+ T cells can be promoted by cytokines into the tumor microenvironment. IFN-γ can reverse functional defects in antigen presentation by increasing the expression of MHC I on tumor cells or immune cells (40). other epitope based IFN-γ stimulating vaccines study also showed a similar role for CD8+ T cells in tumor eradication (41). Cross-presentation is also one of the potential major mechanisms for MHC-II restricted epitopes to induce CD8+ CTL responses, macro-autophagy of the exogenous antigens in tumor cells is essential process for antigen sequestration and delivery to dendritic cells for cross-presentation (42), CD4+ Th cells itself can also modify the APC, convert it into an effective stimulator for the successful cross-priming (43).

One of the most important T-helper cell types in anticancer immunity is Th1 cells. and vaccines that were designed to stimulate tumor antigen-specific T cells, particularly MHC II directed vaccines that promote IFN-γ secretion, could uniquely modulate the tumor microenvironment. Our scRNA-seq studies from tumor-infiltrating T lymphocytes revealed that the E^mut^ Vax not only promotes the generation of Th1 helper CD4+ cells with enhanced function but also induces Th9 and Th17 cells, further cytokine analysis also confirmed the upregulated IL1, IL-4, IL9 and IL17 etc. after E^mut^ peptide restimulation. In our study, peptides were formulated with CFA/IFA adjuvant, which is known to induce a Th1-dominated response in company with Th2 and Th17 responses. No toxicity related to the observed increase in Th17 cells was found. Therefore, more research is needed to determine the role of Th17 in neoepitope-induced immune responses. Additionally, CFA also contains ligands for Toll-like receptors, which is known to stimulate cytokines, such as IL-1β secretion. IL-1β together with IL-4 could induce antitumor Th9 cell differentiation even in the absence of TGFb (44). It is reported that Th9 cells were effective in the elimination of solid tumors (45). Th17 cells may play a dual role in antitumor immunity (37), which are a key player in inflammation and tissue destruction (38). IL-6 appears critical for inhibition of Foxp3+ Treg differentiation and favors the induction of Th17 cells. In this study, we observed IL-6 induction and Tregs downregulation. IL-6 signaling plays a complex role in inflammation and cancer (46). The pro-tumor functions of IL-6 are mostly through IL6-STAT3 axis which triggers up-regulation of target genes responsible for tumor cell survival, the activation of STAT3 could also block the maturation of dendritic cells (DCs) and inhibit T cell function (47). However, increasing evidence supports that IL-6 trans-signaling is a key player in anti-tumor T cell responses. IL6-STAT3 is also known to downregulate Foxp3 expression and promote Tregs to Th17 cells conversion (47), which inhibits the immune suppression in the tumor microenvironment. The plasticity and differential roles of Th17 cells in tumorigenesis have been reported by the previous studies. For example, Th17 cells differentiated by IL-6, IL-23 and IL-1β in the absence of TGF-β express Th1 master regulator T-bet encoding gene Tbx21, secrete IFNƔ, and promote CD8 effector T cell function and tumor regression, whereas Th17 cells generated by IL-6 in the presence of TGF-β secreted protumorigenic IL-10 and expressed CD39 and CD73, promoting the release of immunosuppressive adenosine (48–51). In addition, IL-6 regulates the priming in tumors and promotes lymphocyte proliferation and trafficking, especially for CD8+ lymphocytes (52).

E^mut^ Vax regulated T cells and reshaped other immune cells in the tumor microenvironment towards anti-tumor reactivity without any signs of autoimmunity. E^mut^ Vax skewed macrophages towards the anti-tumor M1 macrophage subset (**Supplementary Figure 2**) and enhanced the proportion of cytotoxic CD27-CD11b+ NK cells while decreasing the immature CD27+CD11b- NK cells (**Supplementary Figure 3**). It is possible that the skewing of these innate immune cells helps to influence or promote the generation of the CD4+ and CD8+ T cell anti-tumor effector cells.

In summary, the current work provides the first proof-of-principle study for the development of an MHC II multi-peptide vaccine against EGFR neoantigens for lung cancer interception. EGFR mutations induce an un-inflamed TME with less T cell infiltration and increased numbers of Tregs, which results in a more suppressive immune environment (53). EGFR inhibition through TKI treatment is known to enhance MHC class I and II antigen presentation, induce more robust infiltration by immune cells and increases local proliferation of T cells in tumors, which leads to increased activation of immune cells and T-cell mediated tumor killing (54, 55). Thus, TKI treatment before (or at the same time) with E^mut^ VAX may potentially offer a better clinical benefit than treatment with TKI after E^mut^ VAX. With further development, this form of precision immunointerception may have the potential to enhance the precision medicine approach to treatment. We demonstrated that prophylactic vaccination can be an efficacious strategy to control disease occurrence and progression, and it has the potential advantage of inducing and mobilizing multiple anti-tumoral immune cell populations in patients with EGFR mutations that are resistant to EGFR TKI treatments.

## Data availability statement

The raw processed single-cell RNA-seq data used in the study are deposited in the NCBI repository, which can be accessed via the BioProject ID PRJNA933186.

## Ethics statement

The animal study was reviewed and approved by All procedures were approved by the institutional animal care and use committee (IACUC). All mice were housed in the Biomedical Resource Center at the Medical College of Wisconsin, Milwaukee, (AA00001807). Written informed consent was obtained from the owners for the participation of their animals in this study.

## Author contributions

MY, JP were responsible for the overall experimental design with input by KP, QZ and BJ. The project was supervised by MY, JP, QZ, BJ, YW, SS, RS, and KP and JP. conducted flow cytometry analysis, JP and QZ assessed anti-cancer efficacy in animal models. DX did the scRNA-seq analysis. The following were responsible for writing, reviewing, and editing the manuscript: JP, QZ, RS, SS, YW, and MY. All authors contributed to the article and approved the submitted version.

## Supplementary material

Supplementary Figure 1 Clustering analysis of scRNA-seq data from tumor-infiltrating immune cells in adjuvant control and E^mut^ Vax treated mice. A. Expression of the marker genes for CD8+ T, CD4+ T, Treg and Tgd cell. B. Expression of the marker genes for NK cells, neutrophils, B cells, macrophages, and dendritic cells.

Supplementary Figure 2 Effects of the E^mut^ Vax treatment on macrophages within tumor-bearing lungs. **(A)**. Canonical macrophage marker expression based on scRNA-seq data. **(B)**. Heatmap of gene expression for the indicated macrophage subsets – M1 (anti-tumor) and M2 (pro-tumor) macrophages. **(C)**. Distribution of tumor-infiltrating M1 and M2 macrophages. **(D)**. Percent changes of the M1 and M2 macrophage subsets across the control and E^mut^ Vax treatment groups.

Supplementary Figure 3 The effects of E^mut^ Vax treatment on NK cells within tumor-bearing lungs. **(A)**. The total NK cells were identified based on the expression of the *Ncr1* gene. **(B)**. Expression of the CD27-CD11b+ NK gene signature and the CD27+CD11b- NK gene signature in total NK cells. **(C)**. Clustering of the total NK cells into CD27-CD11b+ NK and CD27+CD11b- NK cell subsets. **(D)**. Percent changes of CD27-CD11b+ NK and CD27+CD11b- NK cells across the control and E^mut^ Vax treatment groups.

Supplementary Table 1 Peptide sequences target different EGFR mutations.
