# Supplementary figures and images for: Precision immunointerception of EGFR-driven tumorigenesis for lung cancer prevention
Source: Front Immunol. 2023 Feb 17;14:1036563. doi: 10.3389/fimmu.2023.1036563 (PMC9982083; doi:10.3389/fimmu.2023.1036563)

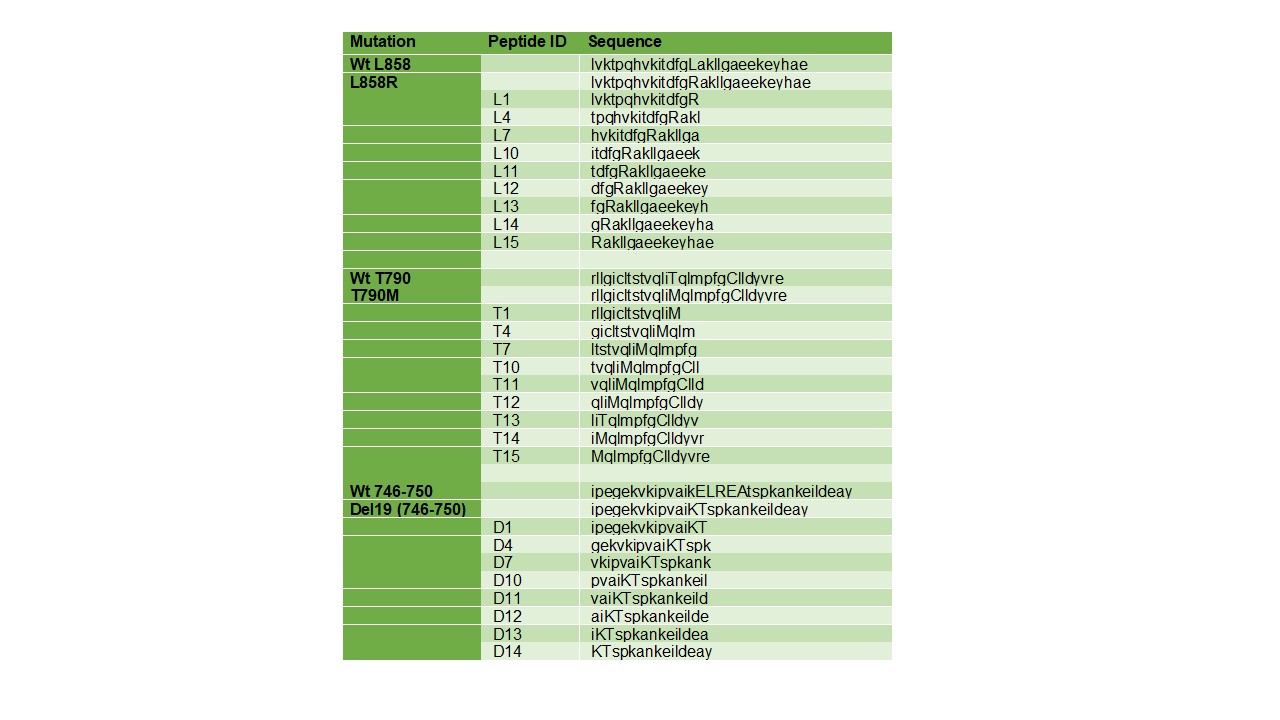

Supplement: Supplementary Figure 1 — Clustering analysis of scRNA-seq data from tumor-infiltrating immune cells in adjuvant control and Emut Vax treated mice. A. Expression of the marker genes for CD8+ T, CD4+ T, Treg and Tgd cell. B. Expression of the marker genes for NK cells, neutrophils, B cells, macrophages, and dendritic cells. [file Image_1.jpeg]

## Slide 1
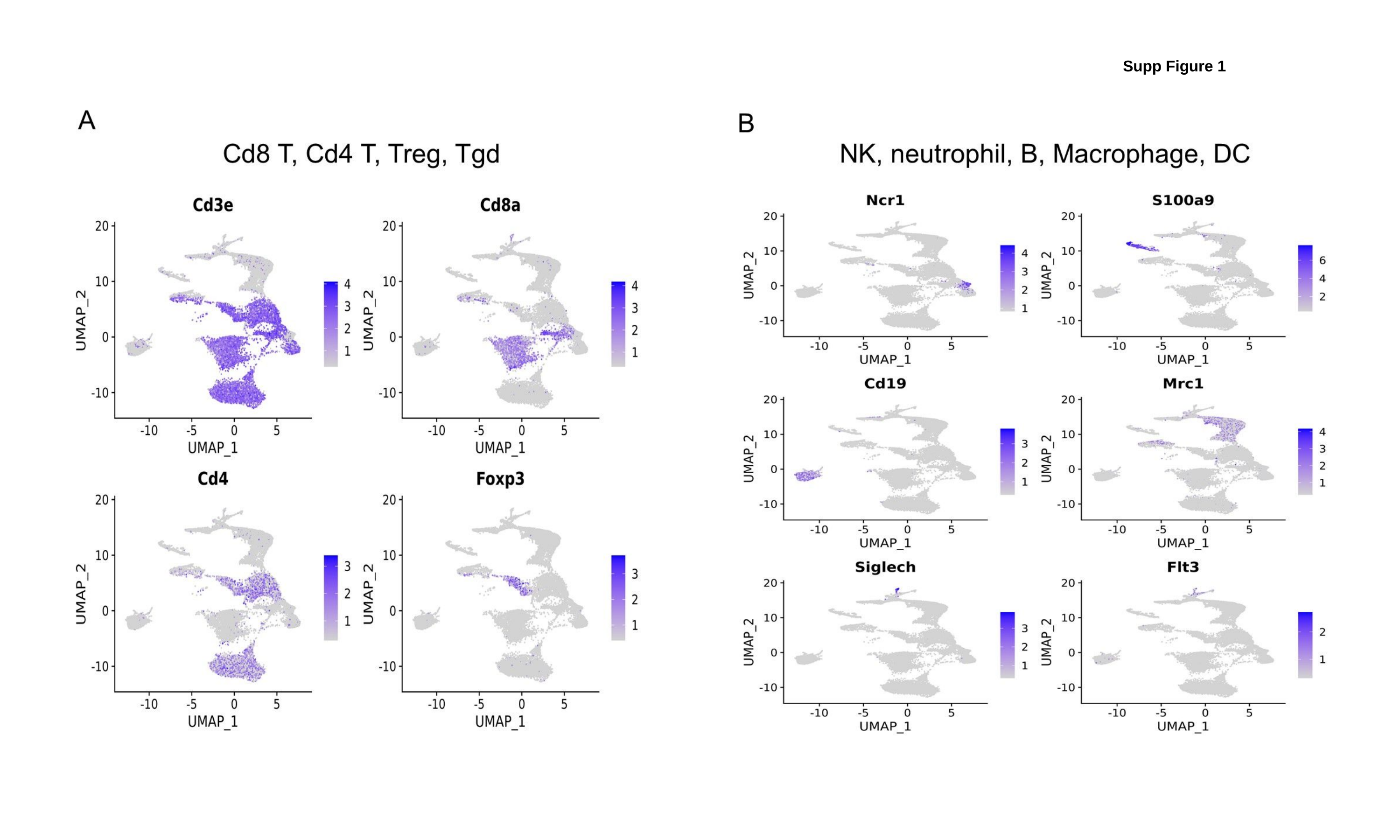

Supp Figure 1

Supplement: Supplementary Figure 2 — Effects of the Emut Vax treatment on macrophages within tumor-bearing lungs. (A). Canonical macrophage marker expression based on scRNA-seq data. (B). Heatmap of gene expression for the indicated macrophage subsets – M1 (anti-tumor) and M2 (pro-tumor) macrophages. (C). Distribution of tumor-infiltrating M1 and M2 macrophages. (D). Percent changes of the M1 and M2 macrophage subsets across the control and Emut Vax treatment groups. [file Presentation_1.pptx]

## Slide 1
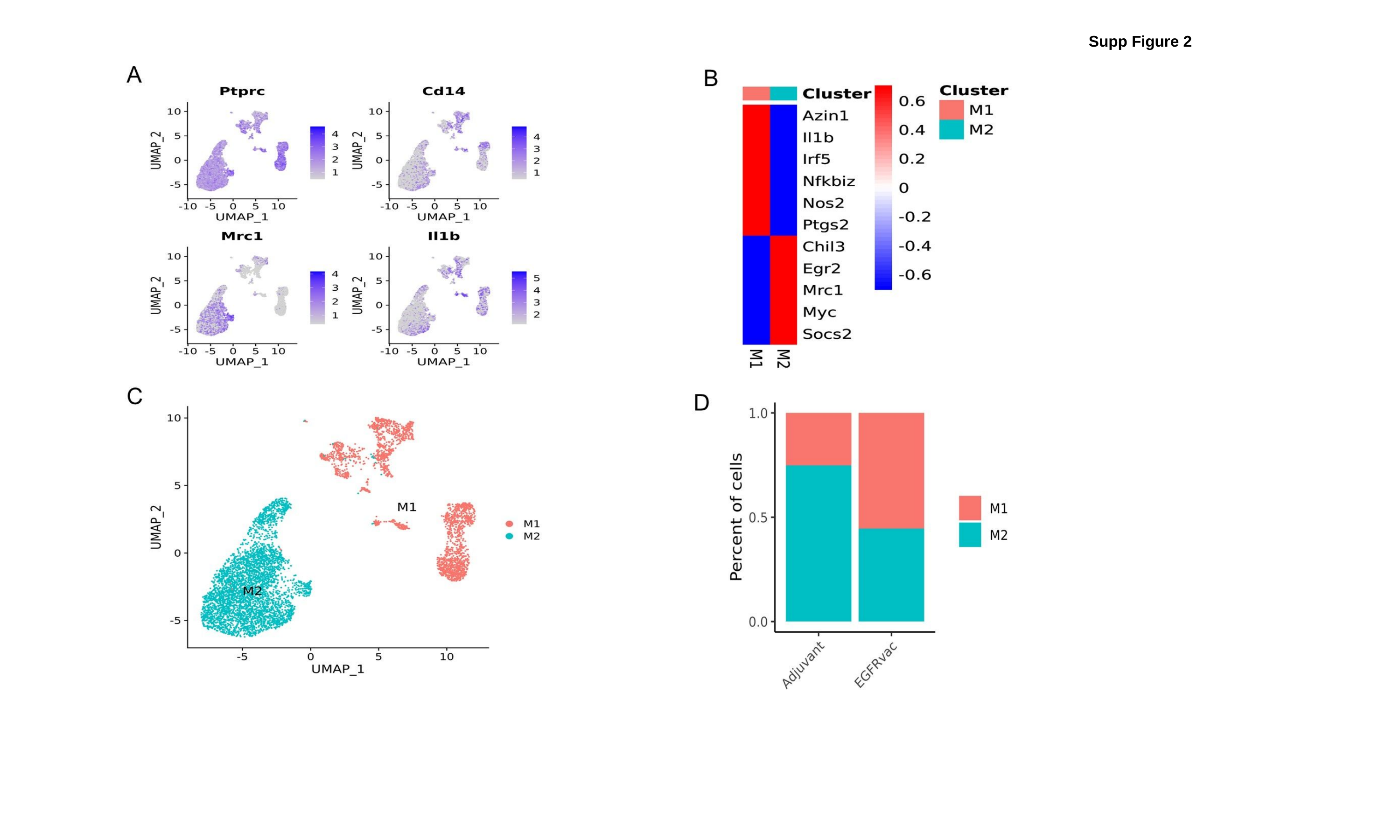

Supp Figure 2

Supplement: Supplementary Figure 3 — The effects of Emut Vax treatment on NK cells within tumor-bearing lungs. (A). The total NK cells were identified based on the expression of the Ncr1 gene. (B). Expression of the CD27-CD11b+ NK gene signature and the CD27+CD11b- NK gene signature in total NK cells. (C). Clustering of the total NK cells into CD27-CD11b+ NK and CD27+CD11b- NK cell subsets. (D). Percent changes of CD27-CD11b+ NK and CD27+CD11b- NK cells across the control and Emut Vax treatment groups. [file Presentation_2.pptx]

## Slide 1
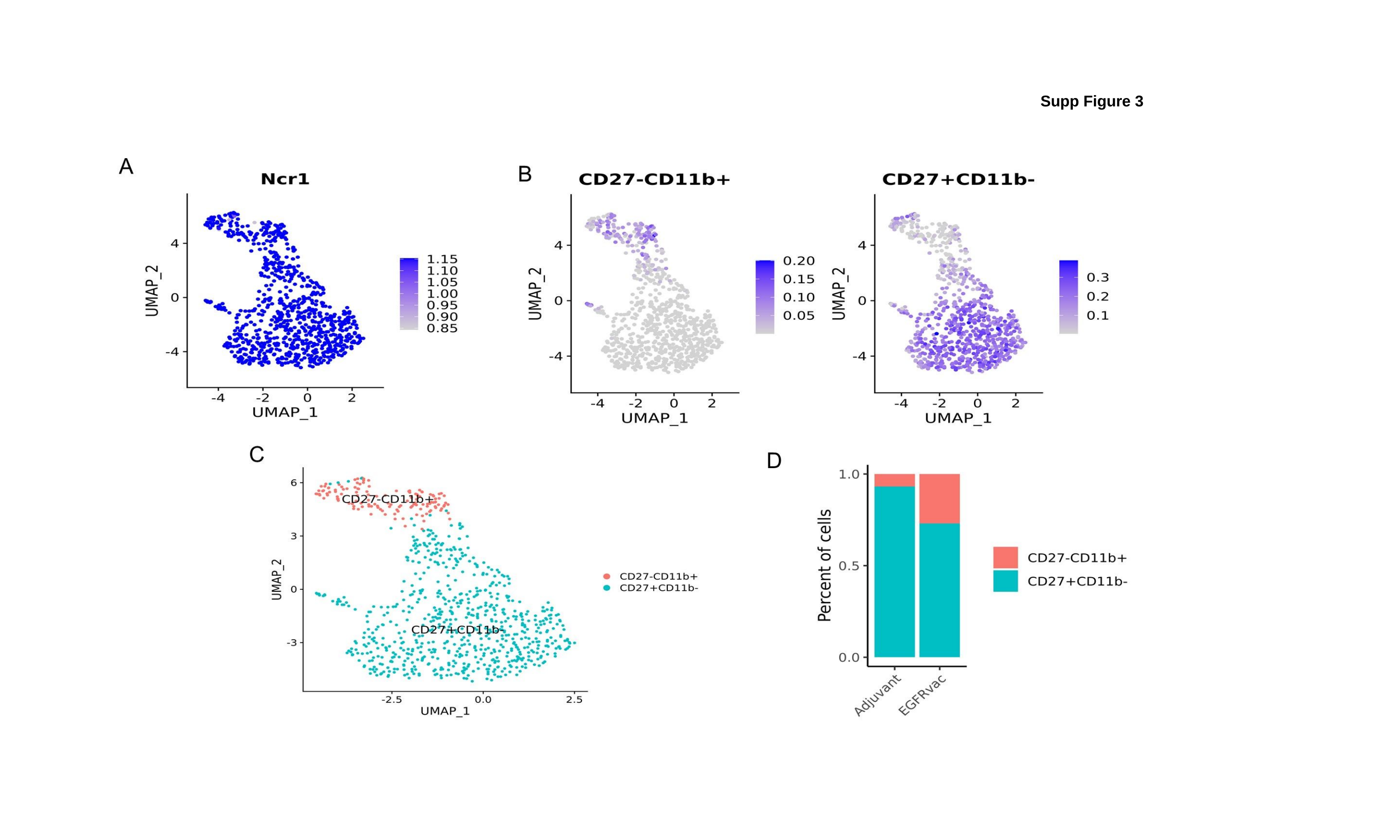

Supp Figure 3

Supplement: Supplementary Table 1 — Peptide sequences target different EGFR mutations. [file Presentation_3.pptx]
